# Rice pyramided line IRBB67 (Xa4/Xa7) homeostasis under combined stress of high temperature and bacterial blight

**DOI:** 10.1038/s41598-020-57499-5

**Published:** 2020-01-20

**Authors:** Gerbert Sylvestre Dossa, Ian Quibod, Genelou Atienza-Grande, Ricardo Oliva, Edgar Maiss, Casiana Vera Cruz, Kerstin Wydra

**Affiliations:** 1 International Rice Research Institute, Los Baños, Philippines; 2 Department of Phytomedicine, Leibniz Universität Hannover, Hannover, Germany; 3 Plant Production and Climate Change, Erfurt University of Applied Sciences, Erfurt, Germany; 4 Present Address: Food and Agriculture Organization, Sub Regional Office for Central Africa, PO. Box 2643, Libreville, Gabon; 5 Present Address: College of Agriculture and Food Science, University of the Philippines, Los Baños, Philippines

**Keywords:** Molecular biology, Plant sciences

## Abstract

Rice bacterial blight (BB) caused by *Xanthomonas oryzae* pv. *oryzae* (*Xoo*) implies substantial yield loss to rice. In times of climate change, increasing temperatures are observed and further acceleration is expected worldwide. Increasing temperature often turns into inhibition of host plant defense to pathogens. Recently, a reduced resistance in rice IRBB4 carrying *Xa4*, but an increase in resistance in IRBB7 carrying *Xa7* resistance by increasing temperature has been reported. Influence of high temperature on both *R* genes (*Xa4+Xa7*) combined in IRBB67 was analyzed under growth chamber conditions and transcriptomic analysis performed. The pyramided line IRBB67 showed no differences in lesion length between both temperature regimes, demonstrating that non-effectiveness of *Xa4* at high temperature did not affect IRBB67 resistance. Moreover, *Xa4* complements *Xa7* resistance with no *Xoo* spread *in planta* beyond the symptomatic area under both temperature regimes in IRBB67. Time course transcriptomic analysis revealed that temperature enhanced IRBB67 resistance to combined heat and *Xoo*. Our findings highlight altered cellular compartments and point at a role of the cell wall involved in *Xoo* resistance and heat stress tolerance in both susceptible (IR24) and the resistant (IRBB67) NILs. Interestingly, up-regulation of trehalose-6-phosphatase gene and low affinity cation transporter in IRBB67 suggest that IRBB67 maintained a certain homeostasis under high temperature which may have enhanced its resistance. The interplay of both heat stress and *Xoo* responses as determined by up-regulated and down-regulated genes demonstrates how resistant plants cope with combined biotic and abiotic stresses.

## Introduction

The world population is projected to reach 9.7 billion by 2050 and half the world population growth is expected in developing countries^1^. This world population growth coupled with the impact of climate change on agricultural production in those countries demands a rapid growth in the food supply and animal feed, while conserving forest resources for climate mitigation^2,3^. Previous reports have shown that an increase of seasonal temperature by 1 °C results in a decline of major grains yield in the range of 2.5 to 16% in the tropics and subtropics^4,5^. Unfortunately, due to their sessile lifestyle, plants have no chance to escape this environment (biotic and abiotic stresses) and must respond and adapt^6–8^.

Abiotic stress may imply positive or negative effects on plant defense responses^7–9^. According to these authors, the outcome of the interaction depends on the timing, nature and the severity of the stress. Temperature, water, relative humidity, light and circadian rhythm significantly influence plant defense and pathogen invasion^6^. A small variation in temperature can affect plant growth, but also plants’ responses to pests and pathogens^10,11^. Most studies on plant responses to environmental changes were carried out under single stress and are therefore unsuccessful in explaining plant responses to more than one stress factor^9^. High temperature affecting host resistance to pathogens has been reported in tobacco infected with Tobacco mosaic virus^12^. Increased disease resistance to stripe rust (*Puccinia striiformis* f.sp *tritici*) was observed in wheat under high temperature (25–35 °C) and is likely caused by the significant expression of resistance gene *Yr36*, which is not effective under low temperature (15 °C)^13,14^. Similar reactions were reported in Arabidopsis, which shows resistance to virulent *Pseudomonas syringae* pv. *tomato* (*Pst*) strain DC3000 at 22 °C^14^ and susceptible to the same strain at moderately elevated temperature between 27–30 °C^14,15^.

In rice, Onaga *et al*.^16,17^ showed that the reaction of resistance genes in rice to *Magnaporthe grisea* under high temperature depends on the genetic background of the rice genotype, where high temperature may either compromise resistance genes’ expression, or enhance the resistance response of gene Pi54. Dossa *et al*.^18^ reported that abiotic stress (drought) resulted in decrease of *Xa4* R-gene mediated resistance to bacterial blight. Studying the effect of high temperature on the rice *Xanthomonas oryzae* pv. *oryzae* (*Xoo*) interaction, Webb *et al*.^19^ reported high temperature reducing the resistance of rice IRBB NILs carrying the *Xa4* resistance gene to *Xoo*. According to these authors, an inverse response was observed in IRBB7, a NIL with *Xa7* resistance gene. This inverse response raised the question about the *R* gene durability under climate change conditions. However, the pyramided lines such as IRBB67 (*Xa4+Xa7*) may be an alternative as pyramided lines are more durable and have broad-spectrum of resistance than monogenic lines^20^. Additionally, a recent study revealed that pyramided line IRBB61 carrying three *R-gene*s (*Xa4*, *xa5* and *Xa7*) showed more resistance to the combined high temperature and *Xoo* stress^21^. Therefore, there is a need to study the molecular mechanisms underlying the IRBB67 carrying two *R-genes* (*Xa4* and *Xa7*) response to the pathogen under high-temperature conditions. The inhibition of plant resistance to pathogens under high temperature is often associated to an enhanced activity of RNA-silencing mediated resistance and an inhibition of effector-triggered immunity under which the pathogen effector is normally recognized by the host *R* gene^22,23^. Recently, a study from Huot *et al*.^24^ reveals that high temperature enhances bacterial type III secretion and disease susceptibility. The resistance induced by the NB-LRR class of *R* gene is reduced by temperature increase due to less nuclear accumulation of SCN1^25^.

The hypothesis here is to determine the effects of *Xa4* on *Xa7* in the pyramided line IRBB67 carrying both *R* genes and to understand how this pyramided line responds to the combination of *Xoo* and heat stress. Combinations pathogen infection with high temperature or with drought stress alter gene expression by activation of specific programs, revealing that plant response to multiple stresses differs between reactions to one or more stresses^26^. Moreover, the study of Rasmussen *et al*.^27^ showed that 61% of transcripts could not be predicted under single stress compared to double stress treatment. Understanding how resistant plants respond to *Xoo* and heat stress will provide information to be used for developing double stress tolerant rice varieties.

## Results

### High temperature affects plant morphology

**Figure 1:**
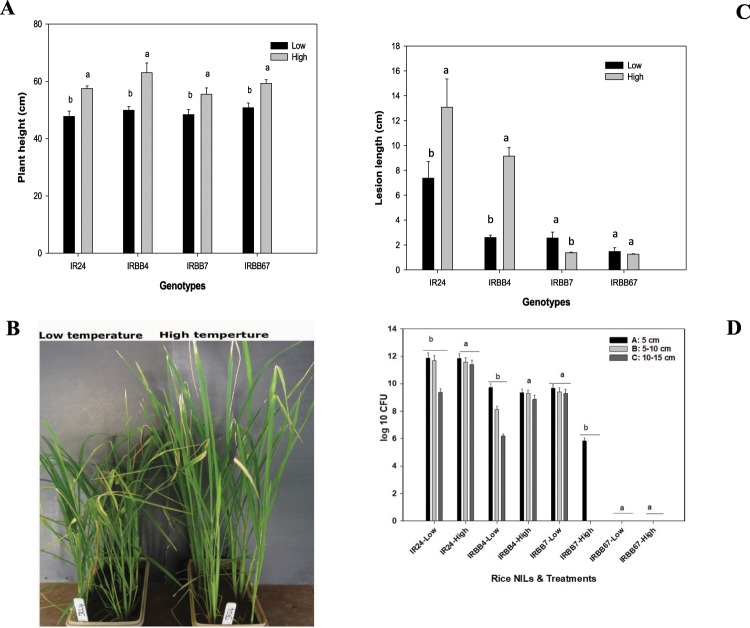
(**A**) Plant height under low and high temperature regimes conditions. Plant height was recorded at 32 days old plants by measuring the length from soil surface to the tip of the most expanded leaf. Plants under high temperature conditions were significantly higher compared to low temperature. The letters a and b represent the significant differences between low and high temperature on each NIL as determined by least significant differences means (LSD means) at α < 0.05. (**B**) Picture of rice NIL IR24 under both temperature regimes. Plants under high temperature conditions were significantly higher compared to low temperature. (**C**) Bacterial blight lesion length under low and high temperature regimes conditions. Bacterial blight lesion length was recorded at 32 days old (11dpi). The letters a and b represent the significant differences between low and high temperature on each NIL as determined by least significant differences means (LSD means) at α < 0.05. (**D**) PXO145 spread *in planta* in four rice NILs under two temperature regimes. Data were collected from leaf clipping inoculated leaves of 21 days old plants and collected 11 days after inoculation. A, B and C represent leaf segments of 5 cm length beyond bacterial blight symptomatic area. The letters a and b represent the significant differences between low and high temperature on each NIL as determined by least significant differences means (LSD means) at α < 0.05.

Plant morphology change due to temperature rise was determined by measuring the plant height under both temperature regimes. All rice NILs were taller under high temperature compared to normal temperature (Fig. 1A). Under low temperature, the plant height ranged from 47.8 cm to 50.8 cm, and under high temperature from 55.5 cm to 63 cm. The highest plant height under low temperature was recorded on IRBB7 and under high temperature on IRBB4 (Fig. 1B).

### Rice near isogenic line with *R* genes *Xa4* and *Xa7* combination confers strong resistance to BB under high temperature

Rice bacterial blight *R* genes *Xa4* and *Xa7* are among the major *R* genes mediating resistance to BB. In order to determine the high-temperature effect on the combination of the two *R* genes (*Xa4* and *Xa7*) responses to BB, rice NIL IRBB67 carrying *Xa4* and *Xa7*, along with IR24 (susceptible) and IRBB4 carrying *Xa4* and IRBB7 carrying *Xa7* were used. Plants were inoculated under both temperature regimes in the growth chamber with PXO145 (*avrXa4+avrXa7*). Disease evaluation after 11 days showed an inverse response between *Xa4* and *Xa7*, with *Xa7* resistance enhanced under high-temperature conditions. However, the gene combination in IRBB67 did not show a significant difference in disease lesion length between high and low temperatures. Under low temperature the average lesion length was 1.5 cm, under high temperature 1.3 cm on IRBB67 (Fig. 1C).

**Figure 2:**
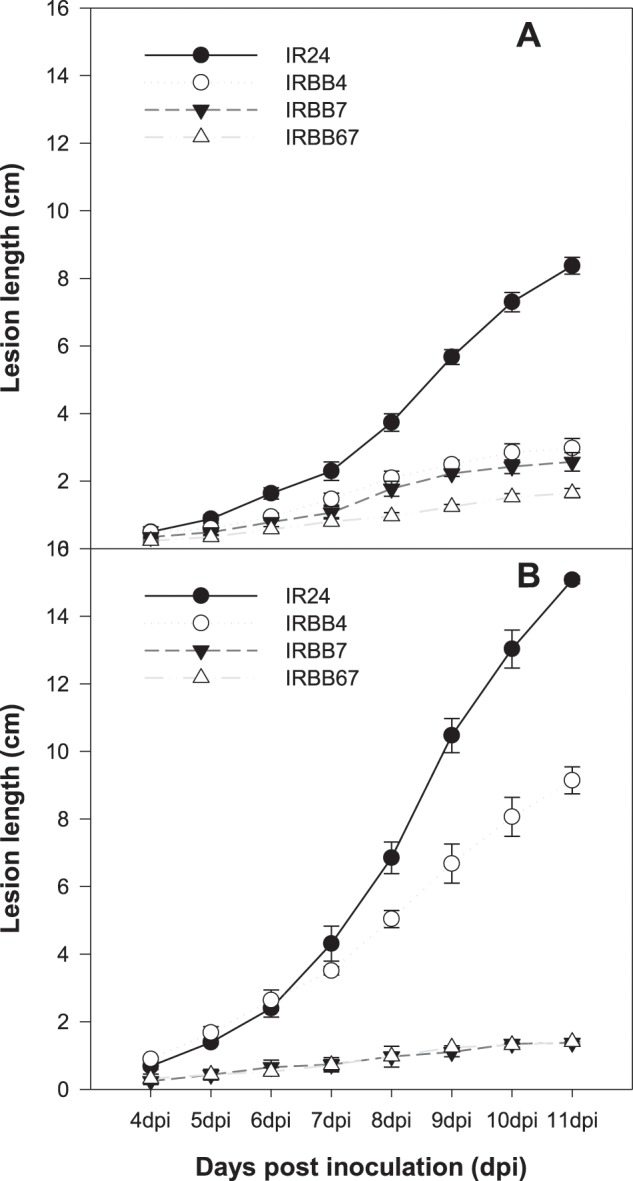
(**A**) Rice NILs IR24, IRBB4, IRBB7 and IRBB67 showing bacterial blight lesion length progression under low temperature regime. (**B**) Rice NILs IR24, IRBB4, IRBB7 and IRBB67 showing bacterial blight lesion length progression under high temperature regime.

Evaluation of bacterial blight lesion length development recorded from 4 dpi to 11 dpi showed that IRBB67 resistance follows a similar pattern to that of IRBB7 under high-temperature conditions (Fig. 2). IRBB67 resistance was high compared to IRBB7 resistance under low-temperature with an average lesion length of 2.6 cm for IRBB7 at 11 dpi. Additionally, *in planta* bacterial counts showed evidence of complementation of *Xa4* to *Xa7* under high-temperature compared to low-temperature (Fig. 1D). The leaf segment plating beyond the visible lesion length showed no significant differences in the bacterial count in IR24 leaves under both temperature regimes. IRBB4 showed similar results at high-temperature while significant differences were observed at low-temperature. However, in IRBB7, the bacterial count between leaf segments was significantly different at high-temperature compared to low-temperature, suggesting that *Xoo in planta* spread less in IRBB7 under high-temperature compared to the spread in IRBB4. Unexpectedly, we did not detect bacterial growth beyond the symptomatic area under both temperature regimes in the pyramided NIL IRBB67.

### Gene expression profiling of IR24 and IRBB67, *Xoo* and mock inoculated under normal and high temperature

To better understand the complementary effects of *Xa4* on *Xa7* response to both bacterial blight and high temperature, total RNA from leaves’ samples of mock-inoculated and bacterial-inoculated plants grown in normal and high temperature conditions were single-end sequenced using Illumina Hiseq technology. The read length of 100 bp in the range of 25.2–82.1 million was generated (Table S2). Percentage of mapped reads of each sample ranged from 97% to 98.6%. The mapped reads were used for further analysis.

**Figure 3:**
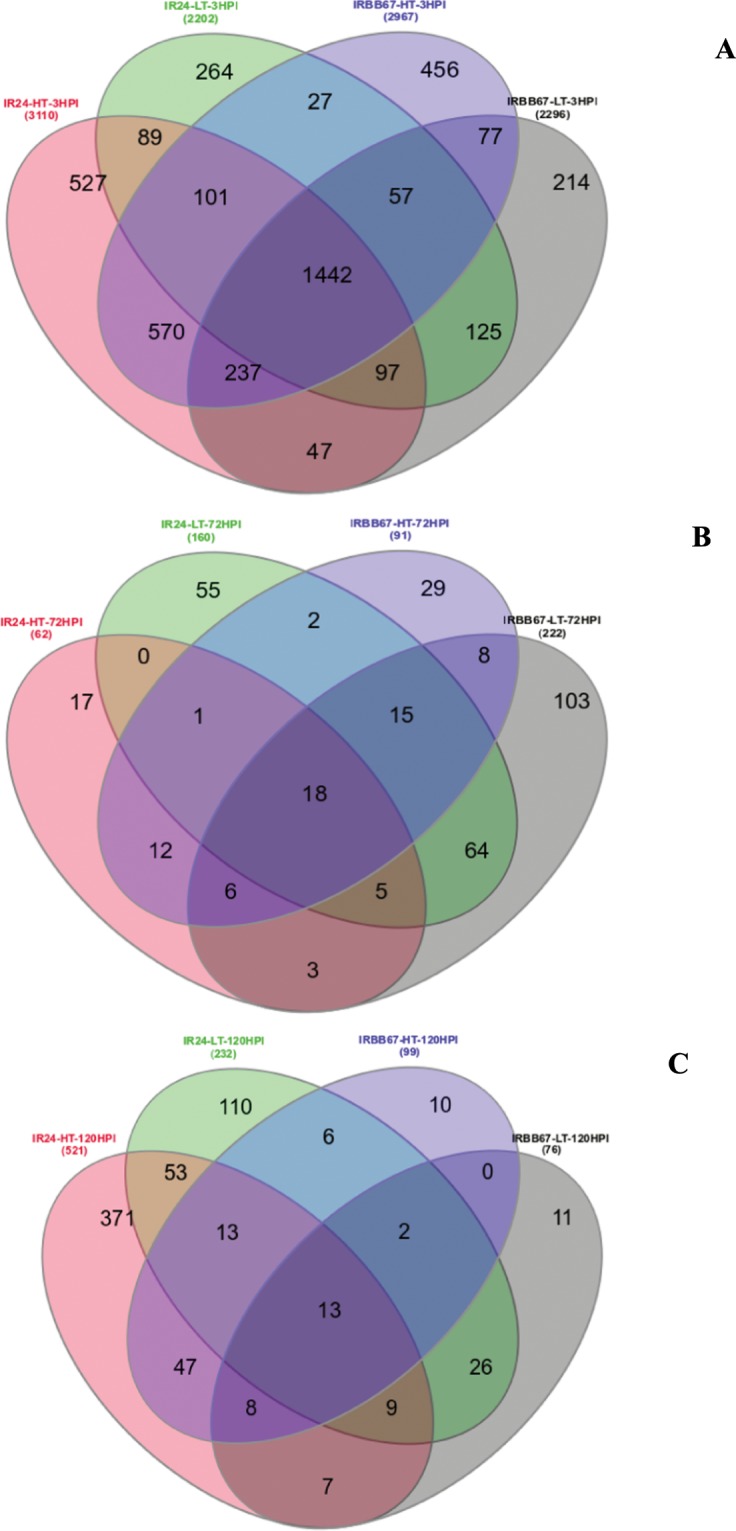
(**A**) Venn diagram showing the distribution of DEGs between *Xoo* and mock inoculated samples under both temperature regimes at 3 hpi. The Venn diagrams were created using jvenny^82^. (**B**) Venn diagram showing the distribution of DEGs between *Xoo* and mock inoculated samples under both temperature regimes at 72 hpi. The Venn diagrams were created using jvenny^82^. (**C**) Venn diagram showing the distribution of DEGs between *Xoo* and mock inoculated samples under both temperature regimes at 120 hpi. The Venn diagrams were created using jvenny^82^.

To identify differentially expressed genes (DEGs), we used the DESeq. 2 package^28^ under R/bioconductor. The significant DEGs were identified based on the false discovery rate (FDR) of 0.05 and log-2 fold change. Using these criteria, we identified 4,683 transcripts differentially expressed comparing *Xoo* inoculated samples to mock inoculated samples from both IR24 and IRBB67 under both temperature regimes at 3, 72 and 120 hpi (Table S3). In IR24, under low-temperature conditions most of the transcripts were differentially expressed at 3 hpi (2,202 DEGs), 160 ones at 72 hpi and 232 DEGs at 120 hpi. In the resistant NIL IRBB67, 2,296, 222 and 76 DEGs were identified at 3, 72 and 120 hpi, respectively, at low-temperature following the same comparison. Under high-temperature conditions, *Xoo* inoculated and mock-inoculated sample comparisons revealed 3,110, 62 and 521 DEGs in IR24 at 3, 72 and 120 hpi, respectively, while in IRBB67, 2,967, 91 and 96 DEGs were identified at the same points (Fig. 3). The temperature increase showed significant effects on DEG numbers, especially at 3 hpi in both IR24 and IRBB67. IRBB67 showed a decrease in DEG numbers with increase in the incubation period (hour post inoculation), suggesting that this rice NIL responds sufficiently to combined stresses of BB and high-temperature at an early stage of infection. Both NILs showed significant reduction in DEGs at 72 hpi, however, IR24 showed more DEGs induced at 120 hpi compared to 72 hpi time points under both temperature regimes, with more DEGs (521) induced at high-temperature conditions.

Functional classification using of the 4,683 transcripts differentially expressed using Pageman analysis^29^, revealed late (120 hpi) up-regulation of hormone metabolism in IR24 at high-temperature while it was induced at 3 and 120 hpi under low-temperature. In IRBB67, the hormone metabolism was up-regulated from 3 to 72 hpi under low-temperature and up-regulated across the three time points under high-temperature conditions. Jasmonate metabolism did not show significant differences in expression between both rice NILs and between both temperature regimes. Additionally, ABA and an ABA induced response were shown to be up-regulated only in IR24 under low-temperature. Under temperature increase conditions, no differences were seen between *Xoo* inoculated and mock-inoculated samples. SA was seen to be up-regulated at 3 and 120 hpi in IR24 under low-temperature and only induced at 72 hpi under high-temperature conditions. In IRBB67, SA up-regulation was observed at 72 hpi under low-temperature and from 72–120 hpi under high-temperature (Table S4).

The cell wall plays several roles such as a physical barrier against insects and pathogens and providing shape and structure but is also involved in cell-cell communication and osmotic regulation. Cell wall biochemistry may play a decisive role in defense response to pathogens, as revealed specifically for bacterial pathogens^30–33^. In this study, the cell wall was generally affected by high-temperature combined with *Xoo*, especially in IRBB67. Similarly, the cell wall proteins such as AGPs (arabinogalactans-proteins) which link the cell wall with the plasma membrane and the cytoskeleton^34,35^ and were described to be involved in defence against bacterial pathogens^30^ were shown to be downregulated by high temperature and *Xoo* in both rice NILs (IR24 and IRBB67) at an early stage of inoculation (Table S4). Further up- and downregulations of cell wall related genes as temperature response were observed. Our results point at an important role of phytohormone regulation and cell wall modifications in response to combined stress of high temperature and bacterial blight, with particularly SA signaling and cell wall proteins likely being important in IRBB67 response to the combined stress.

### IRBB67 regulates several biological processes and molecular functions under high-temperature conditions

To determine the effects of high-temperature conditions on rice, DEGs from mock-inoculated plants at high and low temperature were compared at each time point and within NILs. A total of 332 DEGs were differentially expressed in both mock inoculated NILs. Several DEGs were down-regulated in both NILs in response to high temperature. Differences in rice transcript accumulation between high and low temperatures were observed at 3 hpi at which most of the DEGs were down-regulated (Table S5).

**Figure 4:**
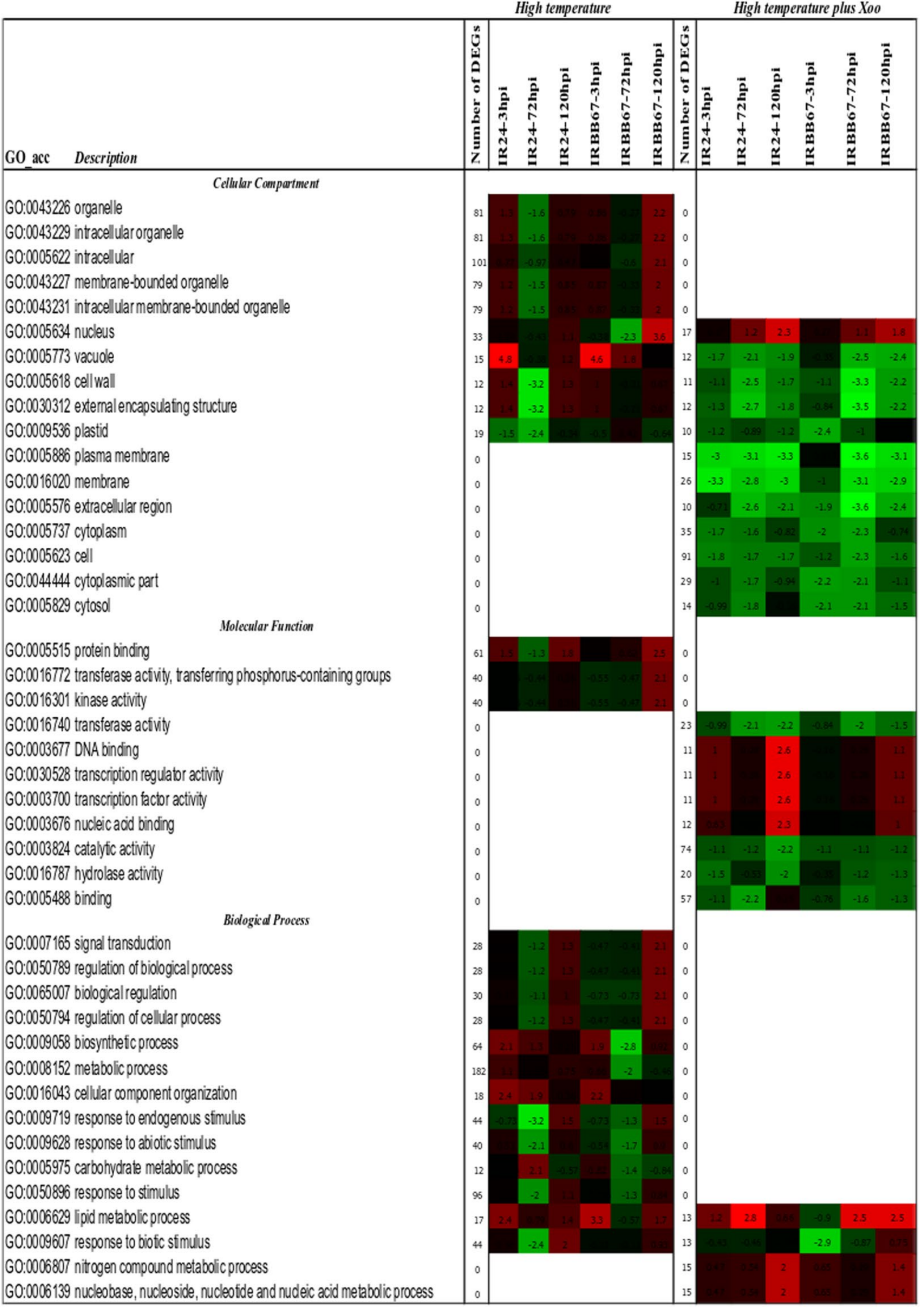
GO enrichment in DEGs from high vs low temperature conditions after mock and *Xoo* inoculation. The enriched GO terms across the three time points and from both NILs (IR24 and IRBB67) as predicted using AgriGO tools Parametric Analysis of Gene Set Enrichment (PAGE) at p-value 0.05^80^.

Functional analysis of the DEGs revealed enrichment in three functional groups (Fig. 4). Twelve DEGs were enriched, deriving from the external encapsulating structure (GO:0030312) and the cell wall (GO:0005618), with down-regulation in IR24 at 72 hpi. The third functional group was nucleus (GO:0005634) and showed overall up-regulation in the resistant reaction at 120 hpi. The DEGs, 32 in total enriched in nucleus at 120 hpi in IRBB67 were significantly down-regulated at 3 hpi.

Besides, 05 DEGs (LOC_Os07g34520.2; LOCOs01g12490.1; LOC_Os08g30020.3; LOC_Os07g34520.3; LOC_Os11g46850.1) were up-regulated in IR24 at 3 hpi and LOC_Os08g04500.2 encoding for terpene synthase was up-regulated at 72 hpi. In the resistant NIL IRBB67, 9 DEGs were up-regulated (Table S5) including MYB family transcription factor (LOC_Os02g53670.1) and MYB transcription factors, which are involved in plant development, but also in defense responses to hormone or stress treatments^36^. Additionally, flavin mono-oxygenase (LOC_Os01g12490.1), which plays an important role in n-tryptophan (Trp)-dependent indole-acetic acid (IAA) biosynthesis in plants and regulates plant growth and development, was among the nine up-regulated DEGs in IRBB67. Taken together, these findings suggest that IRBB67 responds to heat stress by inducing plant growth and development related genes, but also defense response genes.

### High-temperature during pathogen infection affects rice cellular compartments

To explore the effects of high-temperature on rice during pathogen infection, we compared transcript accumulation between high and low temperatures of *Xoo* inoculated samples from both IR24 and IRBB67. A total of 156 DEGs (Table S6) were induced from both IRBB67 and IR24 NILs, demonstrating the repression of several DEGs in response to *Xoo* inoculation between high and low temperatures compared to that of mock-inoculated (Table S5). Besides, only 19 DEGs were shared between mock and *Xoo*-inoculated samples (Fig. S1).

At 3 hpi, no significant DEG was found to be up or down-regulated, except LOC_Os08g39850.2 encoding for lipoxygenase, a chloroplast precursor involved in the programmed cell death pathway and biotic and abiotic stress response in plants^37^, which was down-regulated in IRBB67 (Table S6). At 72 hpi, 14 DEGs were expressed in IR24 with down-regulation of 9 DEGs and up-regulation of 5 DEGs. In the resistant NIL, the difference in the response to *Xoo* between high and low-temperature was generally significant at 72 hpi compared to the susceptible response at the same time point. Fifty four and 39 DEGs were down-regulated and up-regulated, respectively, in IRBB67 at 72 hpi. A late response was observed in the susceptible NIL at 120 hpi, where 63 DEGs were expressed with 7 down-regulated DEGs and 56 up-regulated. Only 5 significant DEGs were expressed in IRBB67 at 120 hpi and all were up-regulated. These 5 DEGs included genes encoding for terpene synthase, putative, expressed (LOC_Os08g04500.1, LOC_Os08g04500.2), methyltransferase, putative, expressed (LOC_Os05g01140.1), Jacalin-like lectin domain containing protein, putative, expressed (LOC_Os12g14440.1), helix-loop-helix DNA-binding domain containing protein, expressed (LOC_Os01g01840.1) and WRKY65, expressed (LOC_Os12g02470.1) (Table S6).

Functional analysis of the 156 DEGs showed down-regulation of plasma membrane (GO:0005886) and membrane (GO:0016020) genes across the three time points in IR24 and at 72 and 120 hpi in IRBB67 (Fig. 4). Genes of the cell wall (GO:0005618), extracellular region (GO:0005576), and external encapsulated structure (GO:0030312) were significantly down-regulated at 72 hpi in both NILs while transferase activity (GO:0016740) was down-regulated in IR24 at 120 hpi, and vacuole (GO:0005773) was down-regulated in IRBB67 at 72 hpi. The late response observed in the susceptible NIL IR24 at 120 hpi was correlated with up-regulation of the following GO terms: transcription regulator activity (GO:0030528), transcription factor activity (GO:0003700), DNA binding (GO:0003677) and nucleic acid binding (GO:0003676) in IR24 at 120 hpi (Fig. 4).

### IRBB67 mediated resistance to bacterial blight under low temperature conditions

To determine the difference in transcript accumulation after *Xoo* inoculation between the resistant (IRBB67) and the susceptible (IR24) NILs, selected DEGs in the IRBB67 and IR24 were compared at each time point. A total of 145 DEGs were differently induced in IRBB67 and IR24 after *Xoo* inoculation under low temperature (Table S7). Among them, 102 DEGs (51 up-regulated and 51 down-regulated) were induced at 3hpi, 40 (six up-regulated and 34 down-regulated) at 72hpi and 85 (37 up-regulated and 48 down-regulated) at 120hpi. DEG numbers decreased at 72hpi, however, a late response to *Xoo* was observed at 120hpi with induction of 85 DEGs. Additionally, 51 DEGs were specifically induced at 3hpi and there were 24 DEGs in common between the 3 time points.

GO enrichment analysis of the DEGs revealed no significant GO terms for p-value of 0.05. However, DEGs (LOC_OS02g40130 and LOC_Os02g40190) which belong to the group of protein kinases related genes, involved in cell death, response to biotic stimulus and response to stress were up-regulated in the resistance NIL (IRBB67). Protein kinases play important roles in activation of plant defense mechanisms and signal transduction. Among the 145 DEGs which are differentially induced in IRBB67 and IR24, 21 DEGs were related to protein kinases. Four(04) LRR type receptor like kinase genes were induced among which three (LOC_Os11g29110, LOCOs_02g40130 and LOC_Os11g29090) were significantly up-regulated in IRBB67 at 3hpi and LOC_Os05g46090 was down-regulated at 120hpi. One DEG encoding for cysteine-rich receptor-like protein kinase (LOC_Os02g12130) was down-regulated in IRBB67 (Table S7). Other types of receptor like kinase such as LOC_Os02g40180, LOC_Os06g38760, LOC_Os06g16300, LOC_Os09g18594, LOC_Os11g07170 and LOC_Os09g19500 were down-regulated in IRBB67.

Additionally, protein kinase genes (LOC_Os05g41950, LOC_Os11g44250) were specifically up-regulated in IRBB67 at 3hpi and a DEG (LOC_Os09g18159) which encodes for light repressible receptor protein kinase, putative expressed was only up-regulated at 120 hpi. Two DEGs encode for wall associated kinases (LOC_Os11g47140 and LOC_Os1146860) and LOC_Os07g03920 which encodes for lectin-like receptor kinase, and LOC_Os06g38650 which encodes for RLKs were significantly up-regulated at 3 and 120hpi in IRBB67 in comparison to IR24 after *Xoo* inoculation under low temperature conditions. LOC_Os11g46850 which encodes for wall associated kinase was significantly up-regulated in IRBB67 only at 3hpi as well as receptor kinase (LOC_Os02g40190) and cyclin-dependent kinase G-1 (LOC_Os02g39010) in IRBB67 (Table S7).

In addition to protein kinases related genes, LOC_Os07g05400 encoding for Ferredoxin-NADP reductase, chloroplast precursor, putative expressed, LOC_Os06g38120 which encodes for low-affinity cation transporter, and LOC_Os06g38110, encoding for expressed protein, were significantly up-regulated at 3hpi in IRBB67. Uncharacterized glycosyl hydrolase Rv2006/MT2062, putative expressed, encoded by LOC_Os09g20390, LOC_Os11g44950 (glycosyl hydrolase family 3 protein) showed significant up-regulation in IRBB67 at 120 hpi, and LOC_Os07g46660, encoding ubiquitin carboxyl-terminal hydrolase domain containing protein expression was up-regulated in IRBB67 from 72–120 hpi, while glycine-rich cell wall protein (LOC_Os03g07270) showed up-regulation at 120 hpi (Table S7).

### High-temperature conditions enhance IRBB67 resistance to bacterial blight

In order to understand the mechanisms by which the resistant rice variety IRBB67 harboring *Xa4* and *Xa7* resistance genes, responds to the combined stress of high temperature and bacterial blight, DEGs from the resistant NIL IRBB67 and the susceptible IR24 after inoculation with *Xoo* under high temperature were compared. A total of 188 transcripts were differentially expressed between IRBB67 and IR24 at 3, 72 and 120 hpi under high-temperature conditions. At 3 hpi, 113 DEGs were expressed with 56 down-regulated and 57 up-regulated. At 72 hpi, 99 transcripts were differentially expressed (44 down-regulated and 55 up-regulated) and 145 DEGs at 120 hpi with 77 down-regulated and 68 up-regulated (Table S8).

**Figure 5:**
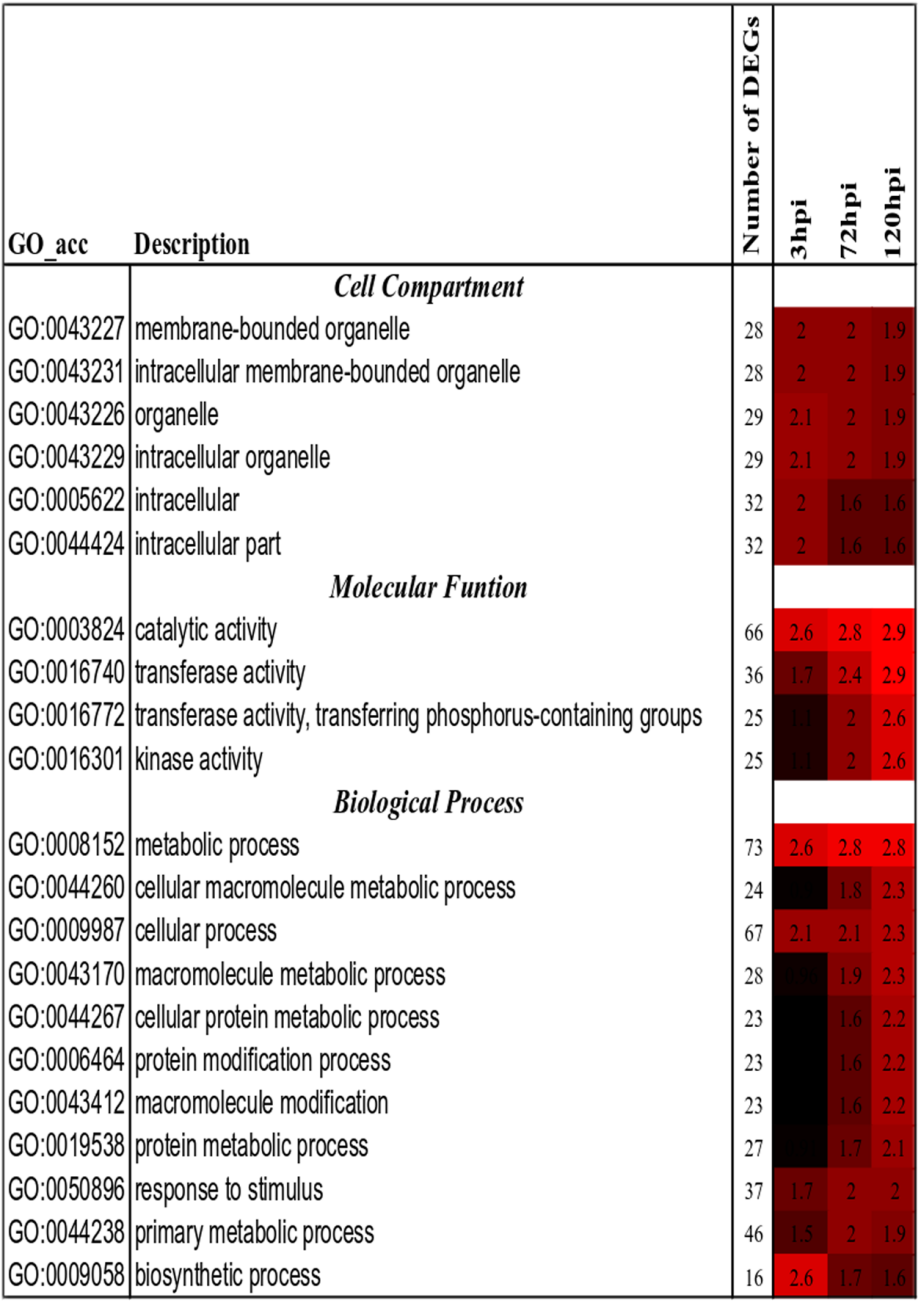
GO enrichment analysis of 188 DEGs expressed between IRBB67 and IR24 under high temperature after *Xoo* inoculation as predicted using AgriGO tools Parametric Analysis of Gene Set Enrichment (PAGE) at p-value 0.05^80^.

Functional analysis of the differential DEGs between IRBB67 and IR24 after inoculation with *Xoo* under high temperature conditions revealed no functional enrichment at 3 hpi, which suggested no response to both high temperature and *Xoo* at an early stage of inoculation from both rice NILs. Examination of GO terms at 72 and 120 hpi suggested that under combined stress of *Xoo* and high temperature, the rice transcriptome is largely devoted to catalytic activity. At 72 hpi, 65 DEGs were functionally enriched in catalytic activity (Fig. 5). Catalytic activity (GO:003824), transferase activity (GO:0016740), kinase activity (GO:0016301) and transferase activity, transferring phosphorus-containing groups (GO:0016772) were the most significant functional groups at 120 hpi. Catalytic activity was shown to be a major regulator in the response to high temperature and *Xoo* in IRBB67, as well as kinase activity (24 DEGs), transferase activity (35 DEGs) and transferase activity and transferring phosphorus-containing groups (24 DEGs) which belong also to catalytic activity groups (Fig. 5).

Besides these four functional groups, DEGs encoding wall associated kinases together with DEGs encoding for low-affinity cation transporter (LOC_Os06g38120.1) and expressed proteins (LOC_Os06g38110.1, LOC_Os06g38210.1, LOC_Os06g38210.2) were significantly up-regulated in IRBB67. Additionally, IRBB67 preferentially responds to the pathogen infection with up-regulation of NB-ARC/LRR disease resistance protein (LOC_Os11g29090.1) and NB-ARC domain containing protein (LOC_Os11g44990.1) and down-regulation of stress response genes such as DEGs encoding for NB-ARC domain containing protein (LOC_Os07g02570.1, involved in stress response, LOC_Os01g24820.1, and LOC_Os11g46210.1, involved in protein binding and plasma membrane). Receptor like kinases function like cell membrane sensors of stimuli and lectin-like receptor kinase 7 (LOC_Os07g03920.1 and LOC_Os07g03970.1) were up-regulated in IRBB67 compared to IR24 in response to high-temperature and *Xoo* (Table S7). DEG LOC_Os09g20390 encoding for uncharacterized glycosyl hydrolase Rv2006/MT2062, putative, expressed involved in trehalose 6 phosphate metabolism, also showed significant up-regulation to combined *Xoo* and high-temperature in IRBB67.

### RNA-Seq validation by RT-PCR

**Figure 6:**
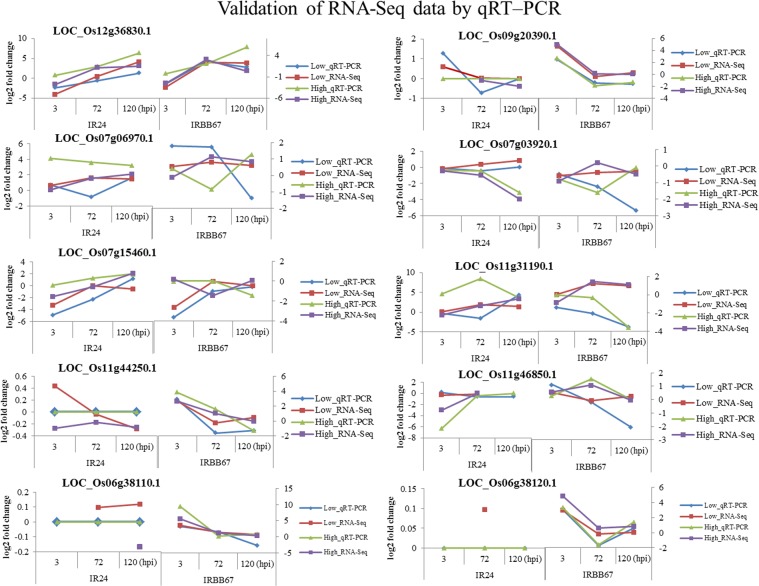
Validation of RNA-Seq data by qRT–PCR.

To validate gene expression pattern determined by RNA-Seq, qRT-PCR was performed on 10 candidate genes (Fig. 6). Expression pattern of the candidate genes determined by qRT-PCR data was in consistent with their expression pattern in RNA-Seq (Fig. 6), although some smaller variation can be seen. LOC_Os11g44250.1, LOC_Os06g38110.1 and LOC_Os06g38120.1 were not induced in IR24 under both temperature regimes confirming the RNA-seq data. The expression of *OsSweet14* (LOC_Os11g31190.1) was much higher in IR24 as determined by qRT-PCR compared to RNA-seq data.

## Discussion

Abiotic stresses are major crop production constraints and are being aggravated by climate change. Under field conditions, plants are exposed to combined abiotic and biotic stresses and the damage induced by several stress factors are more than that of single stress. Increasing temperature affects rice response to BB. Plants have evolved mechanisms to respond to external stimuli such as biotic and abiotic stresses^7,8^ and interactions were described in the rice *Xoo* interaction^38^. Also, virulence factors of the pathogen may play a decisive role in the interaction of bacterial pathogens with host plant components^33,38^. Host plant immunity allows plants to counter-attack the invading pathogen, following pathogen recognition mediated by resistance (*R*) proteins^22^. Our results on the effects of high temperature on the response of rice NIL IRBB67, which pyramids bacterial blight resistance genes (*Xa4+Xa7*), to bacterial blight, revealed a complementation effect of *Xa4* to *Xa7*. *Xa4* appears to be among the most widely used *R* genes in rice breeding programs in Asia^39,40^. As previously reported, *Xa4* resistance to *Xoo* decreases with temperature increase while the inverse trend is seen with *Xa7*^19^. Moreover, the pyramiding NIL IRBB67 harboring the two *R* genes showed resistance to *Xoo* strain PXO145 (*avrXa4+avrXa7*) with no significant differences between both temperature regimes. Also, disease progression recorded on IRBB67 under both temperature regimes is similar to that of IRBB7 under high temperature, suggesting that *Xa4* may not loose completely the resistance and is complemented by *Xa7* in IRBB67. High temperature altered *Xa4* response in IRBB4 to *Xoo*. The mechanisms by which the two *R* genes respond to *Xoo* is still unclear, although it is known that *Xa7* resistance is mediated by *avrXa7* and gives pathogenic fitness cost to the pathogen^41^. Hence, we hypothesized that these two *R* genes use different resistance mechanisms to mediate resistance to *Xoo*. The mechanisms by which *Xa4* triggers the response to the pathogen seems to be affected by temperature rise. In contrast, *Xa7* resistance mechanisms may confer an abiotic tolerance component which enhances its reaction to *Xoo* under temperature rise, therefore, *Xa4* resistance mechanism may not be affected by temperature when it is combined with *Xa7* in IRBB67. This corroborates an early report that high temperature can negatively impact the temperature sensitive resistance to stem rust in oat cultivars harboring Pg3 and Pg4 genes^42^. Moreover, *Xa7-*mediated resistance to *avrXa7* pathogen shows stronger and faster response at high temperature than at low temperature^21^.

*In planta Xoo* spread beyond the symptomatic area suggested that *Xa4* complements *Xa7* at low temperature in IRBB67, thereby reducing *Xoo* spread *in planta*. The predominance of *Xa7* over *Xa4* in IRBB67 mediates strong resistance to PXO145 under high temperature with no *Xoo* spread *in planta* beyond the symptomatic area. High temperature promoting higher bacterial multiplication in the host was reported to possibly depend on the type of *R* gene mediating resistance to the pathogen^43^ as we observed in the current study as inverse reaction between *Xa4* and *Xa7*. Moreover, it is plausible that these two genes may belong to two different classes of genes coding *R* proteins and are modulated by temperature. Consistently, SNC1 gene, a NB-LRR type of *R* gene, does not confer resistance at high temperature when activated compared to low temperature in Arabidopsis^44^, suggesting that *Xa4 R* gene may belong to this type of R gene. Further study will have to investigate this hypothesis since both *R* genes (*Xa4* and *Xa7*) are not cloned and the putative proteins are not known. Wang *et al*.^15^ suggest that high temperature inhibiting plant defense to pathogens may be regulated by defense signalling components or by a combination of multiple factors. Bacteria spread in IRBB4 under high temperature suggests that recognition of corresponding *avrXa4* by *Xa4 R* gene might be compromised by high temperature while effector recognition increased in IRBB7 leads to resistance increase. These results are in contrast to Chen *et al*.^43^, whose findings suggest that high temperature promotes vigorously bacteria multiplication compared to low temperature. Additionally, the rice bacterial blight pathogen during its interaction with the host plant injects effector proteins that bind to host susceptibility genes (*OsSweet*), a sugar transporter gene. Induction of *OsSweet14* caused by TAL effectors *PthXo3*^45^, *avrXa7*^46^, *Tal5*^47^ and *TalC*^48^ results in increase in *Xoo* growth and lesion development. In the resistant plant IRBB67, *OsSweet14* (LOC_Os11g31190.1) was down-regulated, and more significantly at 120 hpi (Table S7).

Examining the gene expression in IR24 and IRBB67 in response to PXO145 and two temperature (high and low) regimes, we found that the differentially expressed genes at early time triggered an efficient response to the pathogen under both temperature regimes in IRBB67. However, in IR24, the pathogen progression *in planta*, enabled by late expression of genes in IR24 under both temperature regimes with more differentially expressed genes under high temperature (Fig. 3). In response to environment changes, cells initiate a gene expression program to adjust its physiology and metabolism to the new environment, preventing it for damage or death^49^. In this study, it appears that at 3 hpi, high temperature and *Xoo* modulate more DEGs in both resistant and susceptible NILs compared to that of low temperature. Phytohormones play important roles in host immunity and response to environmental stimuli^50–53^. In this study, phytohormone related genes were induced, especially SA which is up-regulated in the resistant NIL from 72–120 hpi under high temperature. In contrary to this finding, a similar study revealed repression of SA genes under combined stress of high temperature and bacterial blight in IRBB61 (*Xa4, xa5* and *Xa7*) at 3 and 24hpi^21^, however, in our study, the SA pathway is up-regulated at 72 and 120hpi, suggesting that SA is involved in downstream reactions to sustain the combined *Xa4/Xa7*- mediated resistance to high temperature and bacterial blight. Moreover, one of the major SA signaling regulators (*OsNPR1*, LOC_Os01g09800.1) was significantly up-regulated by the pathogen under high temperature. Expression of SA related genes in both rice NILs appears to be a general response to pathogens, which is enhanced by high temperature in the resistant plant. Salicylic acid has numerously been reported to play an important role in resistance against pathogens^54–60^.

To invade the host organism, pathogens need to encounter the host cell wall, the first physical defense barrier. In case of *Xoo*, it enters the host through hydathodes or wounds^61,62^. The plant cell wall is a battle ground between the pathogens and its host, where highly specific interactions between the pectic polysaccharides homogalacturonan, rhamnogalacturonan I and proteins such as arabinogalactan protein (AGP) and polygalacturonase-inhibiting protein (PGIP) of the cell wall and cell wall degrading enzymes of the pathogen interact, contributing to either an incompatible or a compatible interaction, underlining a decisive role of the plant cell wall in plant defense^30–33^. Further molecular resistance responses^63^ follow, as the information of the foreign invasion is triggering reactions in the nucleus. In this study, the infiltration allowed the bacteria to gain access directly to the host xylem allowing the pathogen to interact directly with the host parenchyma cells^62,64^. It appeared that high temperature increased host colonization by the pathogen as the host cell wall is affected allowing the pathogen to move easily *in planta* when no *R* gene is present. Moreover, the cell wall is a source of nutrients to the pathogen deriving from cellulose, hemicellulose and pectic polysaccharide layers which the pathogen degrades during the infection process^33,65^. The general down-regulation of genes of cell wall metabolism, specifically of genes involved in AGP production and in cell wall degradation in the resistant plants as shown by Pageman analysis (Table S4) points at a role of the cell wall in resistance mediated by this rice NIL under high temperature. However, the comparison between low temperature and high temperature after mock inoculation showed that high temperature generally affects the membrane enclosure with down-regulation of cell wall related genes, external encapsulating structure and plastid related genes in the susceptible NIL IR24, while high temperature in combination *Xoo* repressed the entire cellular compartment in both rice NILs (Fig. 4). Dahal *et al*.^66,67^ also reported the regulation of pathogenesis and stress related and metabolic proteins in interaction with a bacterial pathogen, resulting in suppression of cell wall metabolic proteins in susceptible tomato plants inoculated with *Ralstonia solanacearum*. With the mock inoculation, the nucleus is down-regulated in both IR24 and IRBB67 at 3 and 72 hpi, while a significant up-regulation was seen in IRBB67 at 120 hpi. However, with pathogen stress combined to high temperature, the up-regulation nucleus pathway across the three time points suggests that the host plant stays under permanent alert after pathogen invasion. To sustain the response to this combined stress, lipid metabolism was significantly up-regulated in IRBB67 from 72 to 120 hpi, while the significant expression was observed only at 72 hpi in IR24.

A change caused by high temperature stress on cell wall metabolism is an important physiological mechanism for heat stress tolerance^68^ suggesting that the plant responds by regulating several biological processes and molecular functions under abiotic stress, such as high temperature. This hypothesis could explain the fact that no bacterial spread was detected beyond the symptomatic area in IRBB67 and corroborates the findings from IRBB61 under similar conditions^21^.

High temperature modulated resistance to the pathogen in IRBB67 suggests the existence of a shared pathway between biotic and abiotic stresses. Environmental changes induce plant cells to trigger several events that start with perception of the stimuli at the membrane level^69^. According to the same authors, receptor sensors located in the cell membrane activate several signal transductions that triggered calcium mobilization and other secondary signals to induce stress responsive genes. Calcium acts as second messenger in various stresses^70–73^ and calcium signaling was seen to be up and down regulated in both NILs. However, calcium transport genes (LOC_Os05g02940, LOC_Os03g27960) were up-regulated in IRBB67 at 3 hpi. Additionally, wall associated kinase genes LOC_Os07g03920 and LOC_Os07g03970 were significantly up-regulated in the resistant reaction. Cell walls’ sensing pathogen invasion and high temperature triggering the plant response with up-regulation of receptor kinase gene LOC_Os07g03920 was also reported by Narsai *et al*.^74^ who observed a similar response of the resistant cultivar to bacterial blight. Response to combined high temperature and *Xoo* showed that the resistant NIL devoted large parts of its transcriptome to catalytic activity (Fig. 5). Physiological mechanisms may contribute to IRBB67 resistance to *Xoo* under high temperature. Up-regulation of trehalose phosphate phosphatase gene (LOC_Os09g20390) which dephosphorylates trehalose phosphate synthase to release free trehalose^75^, recently reported to be involved in anaerobic germination in rice^76^ may also play an important role in IRBB67 tolerance or adaptation to high temperature to trigger resistance to *Xoo*. Interestingly, up-regulation of the trehalase gene LOC_Os10g37660 was seen in the resistant NIL, suggesting that conversion of trehalose to glucose for carbohydrate metabolism may contribute to high temperature tolerance in IRBB67. Similarly, a low-affinity cation transporter gene (LOC_Os06g38120) was also up-regulated in the resistant NIL compared to IR24 in response to high temperature and *Xoo*, suggesting a possible role of this gene in maintaining a certain homeostasis of the resistant NIL cell membrane under high temperature. Considering these observations the resistance increase in IRBB7 under high temperature could possibly relate to the genes which are involved in alleviating the high temperature effects on the rice cell membrane by maintaining homeostasis during the stress. Further studies are required to prove the possible role of trehalose-6-phosphate and low-affinity cation transporter genes’ functions in IRBB7 and IRBB67. Stress tolerance accompanied with up-regulation of resistance genes such as NB-ARC/LRR (LOC_Os11g44990) and NR-ARC domain containing protein (LOC_Os11g44990) may contribute to enhance resistance to *Xoo* under high temperature in IRBB67 and possibly in IRBB7, while their activities might be reduced in IRBB4 under high temperature. Hu *et al*.^77^ showed that *Xa4* encodes a cell-wall associated kinase, and strengthening of the cell wall by *Xa4* enhances resistance to bacterial infection. Moreover, genes involved in cell wall modification are down-regulated under high temperature and high temperature combined with *Xoo* inoculation, suggesting that *Xa4* resistance is reduced under heat stress condition. Total resistance activity below the threshold under high temperature results in no defense^25^.

Under low temperature, although no significant enrichment was seen between both rice NILs, up-regulation of defense related genes in the resistance NIL suggests an early recognition of PXO145 in IRBB67 that triggered the defense reaction. Moreover, the up-regulation of the defense genes in IRBB67 in comparison to the susceptible NIL IR24 may suppress pathogen growth and spread *in planta*^78^. Given that trehalose is a universal stress molecule, the up-regulation in the resistant NIL IRBB67 of LOC_Os09g20390 compared to the susceptible suggests that it may be involved in the defense response to *Xoo*.

## Conclusion

In the light of our overall results, high temperature affects the host response to *Xoo*. Time course transcriptome profiles revealed evidence that the resistance of IRBB67 is enhanced under high temperature as several physiological changes were observed in comparison to the susceptible NIL IR24. The response to high temperature with regulation of cell membrane homeostasis might confer high temperature stress tolerance in IRBB67. Complementation effects of *Xa4* affecting *Xoo* spread *in planta* as observed in IRBB67 under both temperature regimes suggest that this *R* gene may not be completely lost under high temperature, and that stress (high temperature) tolerance failure could possibly explain the decrease in *Xa4* effectiveness compared to that of *Xa7* under temperature increase. Our study showed that *R* gene *Xa7* can complement the partly failure of *Xa4* under high temperature, and a combination of both genes is therefore suggested to stabilize resistance of rice to *Xoo* under climate change. Thus, further investigations of how IRBB67 mediates resistance to *Xoo* under high temperature will reveal insight into the crosstalk between the abiotic and biotic stress regulatory pathways.

## Materials and Methods

### Plant growth conditions

Rice NILs’ IR24 (susceptible), IRBB4 (*Xa4*), IRBB7 (*Xa7*) and IRBB67 (*Xa4+Xa7*) seeds were pre-germinated for 4 days at 37 °C on wet filter paper and transferred to pots for further growth under greenhouse conditions (12 h light, 12 h dark). Two-week-old healthy plants were then transferred into indoor growth chambers under two temperature regimes (29/21 °C and 35/31 °C; day/night temperatures) and 70% of relative humidity. Inoculation was conducted on 21 day-old plants.

### Plant inoculation

The inoculum of the Philippines’ *Xoo* race 7, strain PXO145 (*avrXa4+avrXa7*), was prepared from a 3 day-old culture. Twenty-one day-old seedlings of IR24, IRBB4, IRBB7 and IRBB67 were inoculated by the leaf clipping method^78^ while IR24 and IRBB67 plants were inoculated at 3 points of infiltration using syringe needless with PXO145 and with sterilized demineralized water (mock) for RNA-Seq gene expression analysis^79^. The leaf at the second position of the main tiller was inoculated and disease assessment was performed daily from 4 days post inoculation to 11 dpi. Needless syringe inoculated leaves were sampled at 3, 72 and 120 hours post inoculation (hpi). One leaf from 5 plants each for each rice NIL and treatment were taken at each time point and immediately frozen in liquid nitrogen, and stored at −80 °C.

### Total RNA extraction, library construction, and RNA sequencing

Total RNA was extracted from needless syringe inoculated leaves using TRIzol reagent (Invitrogen, Carlsbad, CA, USA) following the manufacturer’s protocol. Total RNA was then treated with DNase (Promega) and quantified using NanoDrop. RNA integrity was checked using an Agilent 2100 Bioanalyzer (Agilent Technologies, Santa Clara, CA, USA). For each time point and condition, two biological replicates were prepared. A single-end fragment library of 100 bp length was generated from cleaned total RNA following instructions of the TruSeq RNA Sample Preparation kit. Cluster generation of the produced libraries was performed using Illumina_ TruSeq SR Cluster Kit v3 - cBot - HS, and sequenced on a HiSeq. 2000 platform (Illumina) with single-end 100-bp reads and submitted to the Microarray and Deep-Sequencing Core Facility of the University Medical Center Göttingen (Germany). Sequence images were transformed with Illumina software BaseCaller to bcl files, which were demultiplexed to fastq files with CASAVA v1.8.2.

### Transcriptome data analysis

The reads of each sample were mapped to the rice genome version 7 of the Rice Genome Annotation Project (RGAP) at MSU using clcbiogenomics workbench v7.0.4 with the following parameters: 2 for mismatch cost, 3 for insertion cost and deletion cost, 0.5 for length fraction and 0.8 for similarity fraction. The expression data for each sample was exported from clcbiogenomics workbench and analyzed in the R/Bioconductor environment loading DESeq. 2^26^, gplot, ggplot2 packages. The differentially expressed genes were analyzed based on a generalized linear model likelihood ratio test assuming negative binomial data distribution via DESeq. 2. Candidate genes were filtered to a minimum of 4X fold change and FDR-corrected p-value <0.05. Functional association enrichment analysis was conducted following the methodology of Du *et al*.^80^. RNA-Seq data’s (raw read and mapped files) are available through the NCBI Gene Expression Omnibus with accession number GSE79011.

### Time course quantitative real-time PCR validation

Quantitative RT-PCR using SYBR Green detection reagents in Step One Plus (Applied Biosystems, USA) was used to validate the expression of ten candidate genes shown to be differentially regulated from RNA-Seq data. Total RNA from the third biological replicate was used according to each time point. The sequence of each gene was obtained from the Rice Genome Annotation Project database, RGAP7 (http://rice.plantbiology.msu.edu/), and the sequences of genes were used for primers design using qPCR Assay Design tool of Integrated DNA Technology (IDT, http://sg.idtdna.com/site). Rice actin gene was selected as a reference gene in qRT-PCR. 2^−∆∆Ct^ method as described by Schmittgen and Livak^81^ and used to determine the relative expression, and the log2 transformation of the relative expression was used to compare with the RNA-Seq data. All samples were studied in triplicate PCR. The primers and corresponding sequences are listed in Table S1.

## Supplementary information

Supplementary file.

Supplementary dataset.

## Data Availability

The RNA-Seq data (raw read and mapped files) are available through the NCBI Gene Expression Omnibus with accession number GSE79011.
