# Supplementary material for: Rice pyramided line IRBB67 (Xa4/Xa7) homeostasis under combined stress of high temperature and bacterial blight
Source: Sci Rep. 2020 Jan 20;10:683. doi: 10.1038/s41598-020-57499-5 (PMC6971257; doi:10.1038/s41598-020-57499-5)
Supplement: Supplementary file 1 — Supplementary file. [file 41598_2020_57499_MOESM1_ESM.docx]

***Rice pyramided line IRBB67 (Xa4/Xa7) homeostasis under combined stress of high temperature and bacterial blight***

Gerbert Sylvestre Dossa^1,2*ǂ^, Ian Quibod^1^, Genelou Atienza-Grande^1#^, Ricardo Oliva^1^, Edgar Maiss^2^, Casiana Vera Cruz^1^, Kerstin Wydra^2,3^

1: International Rice Research Institute, Los Baños, Philippines

2: Department of Phytomedicine, Leibniz Universität Hannover, Hannover, Germany

3: Plant Production and Climate Change, Erfurt University of Applied Sciences, Erfurt, Germany

ǂ: Current address: Food and Agriculture Organization, Sub Regional Office for Central Africa, PO. Box 2643, Libreville, Gabon

#: Present address: College of Agriculture and Food Science, University of the Philippines Los Baños, Philippines.

* Corresponding author: Gerbert S. Dossa [g.dossa](mailto:c.dossa@irri.org)@hotmail.com

Table S1: qRT-PCR primers for RNA-Seq data confirmation

| **S/N** | **Gene** | **Description** | **F or R** | **Sequence (5’ 3’)** | **Remarks** |
| --- | --- | --- | --- | --- | --- |
| 1 | LOC_Os11g44250 | protein kinase, putative, expressed | F | GCGTTATAGCAGGCACTCTAA |  |
|  |  |  | R | CCCTCTTGCTCACATTCTTCT |  |
| 2 | LOC_Os11g46850 | wall-associated kinase, putative | F | TCGACTGCAACCATAGCTTTAC |  |
|  |  |  | R | GCTGGATTCCGTGGTGTTAG |  |
| 3 | LOC_Os07g03920 | lectin-like receptor kinase 7, putative | F | GTGAGAAGAAGGCTGAGGTATG |  |
|  |  |  | R | CAGTGCCCAAGAGATGACTATT |  |
| 4 | LOC_Os07g06970 | HEN1, putative, expressed | F | CAGTACAGTTGGATCGCTTTCT |  |
|  |  |  | R | CACCACCAAGGAAGCAGTATAG |  |
| 5 | LOC_Os06g38120 | Low affinity cation transporter | F | TTCCTCGCCTTCTCATCTTTC |  |
|  |  |  | R | GTATTGTCAGCACCGGTAGAA |  |
| 6 | LOC_Os12g36830 | pathogenesis-related Bet v I family protein, putative, expressed | F | CAACGCAGCTCACATTATCAAG |  |
|  |  |  | R | CGAGCTCATACTCCACGTTTAT |  |
| 7 | LOC_Os09g20390 | OsTTP6 | F | AACAAGGGAGTCCTCTTCCAG | Kretzschmar et al., 2015 |
|  |  |  | R | CTTGAACGCGTCCTCGTC |  |
| 8 | LOC_Os06g38110 | Expressed protein | F | CGCCGTTCTAATGGACTACTT |  |
|  |  |  | R | AAGGTTTGCGCGGATAGAG |  |
| 9 | LO_Os07g15460 | metal transporter Nramp6, putative, expressed | F | ATGGGGGTGACGAAGGCGGA |  |
|  |  |  | R | ATTTCCAGGATCGAGGTAA |  |
| 10 | LOC_Os11g31190 | OsSweet14 | F | CCTAGGCAACATCATCTCCT |  |
|  |  |  | R | CGATGTAGATGGTCTCGATG |  |
| 11 | Actin |  | F | TCCATCTTGGCATCTCTCAG |  |
|  |  |  | R | GTACCCTCATCAGGCATCTG |  |

F: Forward sequence, R: Reverse sequence. Primers were designed using qPCR Assay Design tool of Integrated DNA Technology (IDT, <http://sg.idtdna.com/site>).

**Table S2: Mapping results of IR24 and IRBB67 (*Xa4/Xa7*) RNA sequencing reads at 3, 72 and 120 hours post-inoculation (hpi) with *Xoo* strain PXO145 (*avrXa4*/*avrXa7*) and water inoculation under two temperature regimes (low and high)**

| **Samples** | | | | **Biological replication I** | | | **Biological replication II** | | |
| --- | --- | --- | --- | --- | --- | --- | --- | --- | --- |
|  | **Temperature treatment** | **Inoculation treatment** | **Time Points** | **Total reads** | **Total mapped reads** | **Percentage of mapped reads (%)** | **Total reads** | **Total mapped reads** | **Percentage of mapped reads (%)** |
| IR24 | Low | Mock | 3hpi | 40,674,840 | 39,746,811 | 97.72 | 45,901,604 | 44,948,818 | 97.92 |
| IR24 | Low | Mock | 72hpi | 43,936,016 | 42,991,838 | 97.85 | 40,369,096 | 39,554,307 | 97.98 |
| IR24 | Low | Mock | 120hpi | 41,557,685 | 40,785,941 | 98.14 | 35,775,914 | 34,862,571 | 97.45 |
| IR24 | High | Mock | 3hpi | 38,816,251 | 38,023,319 | 97.96 | 42,282,183 | 41,385,534 | 97.88 |
| IR24 | High | Mock | 72hpi | 42,608,972 | 41,630,584 | 97.7 | 25,243,662 | 24,644,259 | 97.63 |
| IR24 | High | Mock | 120hpi | 37,366,180 | 36,536,207 | 97.78 | 33,906,037 | 33,209,507 | 97.95 |
| IR24 | Low | *Xoo* | 3hpi | 36,569,942 | 36,056,833 | 98.6 | 34,521,855 | 33,927,051 | 98.28 |
| IR24 | Low | *Xoo* | 72hpi | 38,381,083 | 37,500,798 | 97.71 | 33,215,355 | 32,452,033 | 97.7 |
| IR24 | Low | *Xoo* | 120hpi | 35,997,261 | 35,273,959 | 97.99 | 33,420,582 | 32,638,429 | 97.66 |
| IR24 | High | *Xoo* | 3hpi | 49,697,688 | 48,549,597 | 97.69 | 35,330,289 | 34,660,833 | 98.11 |
| IR24 | High | *Xoo* | 72hpi | 34,472,139 | 33,821,975 | 98.11 | 37,533,718 | 36,416,101 | 97.02 |
| IR24 | High | *Xoo* | 120hpi | 29,264,471 | 28,384,794 | 96.99 | 34,483,333 | 33,778,667 | 97.96 |
| IRBB67 | Low | Mock | 3hpi | 39,041,035 | 38,174,907 | 97.78 | 47,598,258 | 46,647,930 | 98 |
| IRBB67 | Low | Mock | 72hpi | 44,081,764 | 43,241,647 | 98.09 | 41,233,115 | 40,359,389 | 97.88 |
| IRBB67 | Low | Mock | 120hpi | 41,579,556 | 40,605,006 | 97.66 | 30,738,084 | 30,064,125 | 97.81 |
| IRBB67 | High | Mock | 3hpi | 44,707,371 | 43,700,382 | 97.75 | 42,754,098 | 41,869,626 | 97.93 |
| IRBB67 | High | Mock | 72hpi | 34,623,159 | 33,908,453 | 97.94 | 82,171,104 | 80,174,653 | 97.57 |
| IRBB67 | High | Mock | 120hpi | 34,143,236 | 33,458,910 | 98 | 33,053,013 | 32,324,261 | 97.8 |
| IRBB67 | Low | *Xoo* | 3hpi | 39,473,668 | 38,738,159 | 98.14 | 30,236,187 | 29,710,824 | 98.26 |
| IRBB67 | Low | *Xoo* | 72hpi | 34,082,657 | 33,333,817 | 97.8 | 40,133,169 | 39,031,327 | 97.25 |
| IRBB67 | Low | *Xoo* | 120hpi | 35,648,982 | 34,912,253 | 97.93 | 31,983,452 | 31,327,119 | 97.95 |
| IRBB67 | High | *Xoo* | 3hpi | 32,953,631 | 32,346,294 | 98.16 | 29,323,127 | 28,745,107 | 98.03 |
| IRBB67 | High | *Xoo* | 72hpi | 36,696,325 | 35,951,404 | 97.97 | 34,852,161 | 34,101,327 | 97.85 |
| IRBB67 | High | *Xoo* | 120hpi | 25,638,636 | 24,991,603 | 97.48 | 28,561,396 | 27,864,949 | 97.56 |


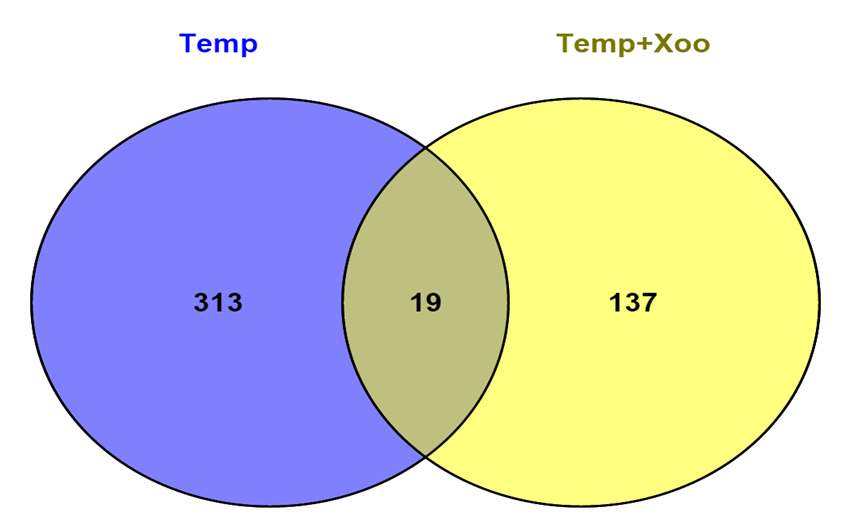


Figure S1: Venn diagram of Differential expressed genes (DEGs) induced under high temperature conditions (Temp) and high temperature plus bacterial inoculation conditions (Temp+*Xoo*)
